# Bile Acids: Key Players in Inflammatory Bowel Diseases?

**DOI:** 10.3390/cells11050901

**Published:** 2022-03-05

**Authors:** Aicha Kriaa, Vincent Mariaule, Amin Jablaoui, Soufien Rhimi, Hela Mkaouar, Juan Hernandez, Brice Korkmaz, Adam Lesner, Emmanuelle Maguin, Ali Aghdassi, Moez Rhimi

**Affiliations:** 1 Microbiota Interaction with Human and Animal Team (MIHA), Micalis Institute, AgroParisTech, Université Paris-Saclay, INRAE, 78350 Jouy-en-Josas, France; aicha.kriaa@inrae.fr (A.K.); vincent.mariaule@inrae.fr (V.M.); amin.jablaoui@inrae.fr (A.J.); soufienrhimi@yahoo.fr (S.R.); hela.mkaouar@yahoo.com (H.M.); emmanuelle.maguin@inrae.fr (E.M.); 2 Oniris, Department of Clinical Sciences, Nantes-Atlantic College of Veterinary Medicine and Food Sciences, University of Nantes, 101 Route de Gachet, 44300 Nantes, France; juan.hernandez@oniris-nantes.fr; 3 INSERM UMR-1100, “Research Center for Respiratory Diseases”, University of Tours, 37032 Tours, France; brice.korkmaz@inserm.fr; 4 Faculty of Chemistry, University of Gdansk, Uniwersytet Gdanski, Chemistry, Wita Stwosza 63, PL80-308 Gdansk, Poland; adam.lesner@ug.edu.pl; 5 Department of Medicine A, University Medicine Greifswald, 17489 Greifswald, Germany; ali.aghdassi@med.uni-greifswald.de

**Keywords:** inflammatory bowel diseases, gut microbiota, bile acids, microbiome, holobiont

## Abstract

Inflammatory bowel diseases (IBDs) have emerged as a public health problem worldwide with a limited number of efficient therapeutic options despite advances in medical therapy. Although changes in the gut microbiota composition are recognized as key drivers of dysregulated intestinal immunity, alterations in bile acids (BAs) have been shown to influence gut homeostasis and contribute to the pathogenesis of the disease. In this review, we explore the interactions involving BAs and gut microbiota in IBDs, and discuss how the gut microbiota–BA–host axis may influence digestive inflammation.

## 1. Introduction

Inflammatory bowel diseases (IBDs), including both Crohn’s disease (CD) and ulcerative colitis (UC), are disabling chronic immune-mediated disorders that have been increasing worldwide [1]. The etiology of IBDs remains elusive but several factors are known to contribute to its pathogenesis, including genetic predispositions, environmental triggers, intestinal immune dysregulation and gut dysbiosis [2]. The latter has been highlighted by several studies that noted reduced microbial diversity and differences in the relative abundances of specific bacterial taxa in IBD patients compared with healthy subjects [3]. The composition of the gut microbiota is known to vary between individuals, reflecting the impact of environmental factors as well as disease states. As a result of IBD-related dysbiosis, the production of bacterial enzymes—and thus, bile acid (BA) metabolism—can be impaired [4]. BAs are the end products of cholesterol metabolism that are synthesized in the liver and secreted into the duodenum through the bile flow [5]. Following synthesis, cholic acid (CA) and chenodeoxycholic acid (CDCA), two major primary BAs, are conjugated to either taurine or glycine and then secreted into the bile. As the BAs reach the terminal ileum, they are reabsorbed by enterocytes and reach the liver via the portal vein, where they are taken up and recycled. BAs are well-known for promoting dietary lipid absorption due to their micelle-forming properties and are believed to play a role in antibacterial defense, influencing both host metabolism and immune responses [6]. They have also emerged as signaling ligands for multiple receptors, including the nuclear hormone farnesoid X receptor (FXR), Takeda G protein receptor 5 (TGR5) and vitamin D receptor (VDR) [7]. These receptors play essential roles in shaping host immune responses. Primary bile acids, including CA and CDCA, as well as their potent secondary BAs have been shown to modulate RORγ+ Treg cells, which are critical in regulating intestinal inflammation through the VDR [8,9]. Lately, the interaction between BAs and gut microbiota in digestive inflammation has drawn considerable attention. Several studies have detected increased levels of primary conjugated BAs in the stool of IBD patients in both remission and an active disease state whereas those of microbially transformed ones decreased dramatically compared with healthy subjects [10]. Similar differences in the composition of BAs have been reported in other studies exploring fecal metabolite pools in IBD patients [11,12]. As the digestive tract and its microbiota are perceived to be a key organ at the crossroads of immune and metabolic processes, we focus herein on investigating the role of BAs in IBDs. In this review, we explore the potential contribution of BAs at the interface between the host immune system and gut microbiota and discuss the mechanistic relevance of BA dysregulation in IBDs.

## 2. Methods

Studies that aimed to understand the relevance of bile acids in digestive inflammation are portrayed in this review. Specifically, to obtain an overview of the research in this area and results achieved so far, we carefully reviewed the literature available on PubMed. The main keywords used for this analysis included microbiome, bile acids, IBD and signaling. After a detailed analysis by at least two co-authors, 74 papers were found to focus on gut microbiome–bile acid interactions in IBDs, which is the main emphasis of this review. 

## 3. Bile Acid Metabolism

**Figure 1:**
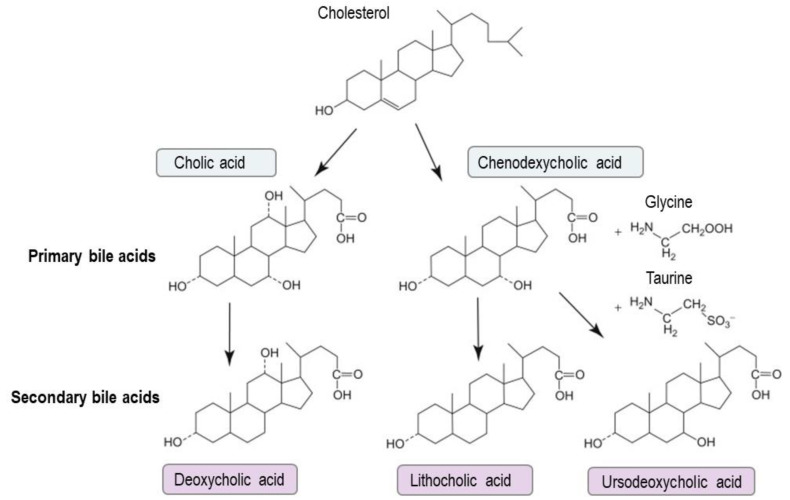
Bile acid synthesis. In the liver, bile acid metabolism mainly produces two primary bile acids, cholic acid and chenodeoxycholic acid. In the intestine, primary bile acids serve as substrates for microbial metabolism by the gut microbiota to generate secondary bile acids, including deoxycholic acid, lithocholic acid and ursodeoxycholic acid.

Bile acids are exclusively synthesized by hepatocytes through cytochrome P450-mediated cholesterol oxidation, a process that follows two pathways—the classic and the alternative pathways [13,14]. The classic pathway is initiated by cholesterol 7α-hydroxylase (CYP7A1), the rate-limiting step that generates the primary CA and CDCA [5,15,16]. The alternative pathway is initiated by mitochondrial sterol 27-hydroxylase (CYP27A1) and produces CDCA [17]. In the liver, most BAs are conjugated to either glycine or taurine via bile acid:CoA synthetase (BACS) and bile acid-CoA:amino acid N-acyltransferase (BAAT) prior to their secretion into the bile (Figure 1). Sulfated or glucuronidated BAs, catalyzed by sulfotransferase family 2A member 1 (SULT2A1) and uridine 5′-diphospho (UDP)-glucuronosyltransferase (UGT) enzymes, respectively, are also amidated with taurine or glycine and finally released into the bile by an efflux transporter called multidrug resistance-associated protein 2 (MRP2). Following their secretion, the conjugated BAs are stored in the gallbladder, thus forming bile with phospholipids, cholesterol and other components [18]. After each meal, the physiological contraction of the gallbladder releases BAs into the duodenum, where they form micelles with cholesterol and dietary fats to facilitate their solubilization and absorption [19,20]. In the ileum, BAs are actively reabsorbed and transported back to the liver, a process termed enterohepatic circulation [13,15,21]. Unabsorbed BAs (5%) that escape the enterohepatic circulation are further metabolized by the gut microbiota [18,21,22] (Figure 1). One important function of the human gut microbiota is the deconjugation of primary BAs and their subsequent biotransformation into secondary BAs. The major bacterial genera involved in such reactions include *Bacteroides*, *Clostridium*, *Bifidobacterium* and *Lactobacillus* for the deconjugation of taurine- and glycine-conjugated BAs into their respective unconjugated forms by bile salt hydrolase (BSH) enzymes [5]. All members of the Lachnospiraceae and Ruminococcaceae families execute the subsequent 7α-dehydroxylation of the deconjugated BAs to generate deoxycholic acid (DCA) and lithocholic acid (LCA), the two most prevalent secondary BAs. Additionally, *Bacteroides*, *Clostridium*, *Escherichia* and *Eubacerium* perform the C7β epimerization of CDCA to generate the 7β epimer, i.e., the 3α-, 7β-dihydroxy-5β-cholanoic acid, also known as ursodeoxycholic acid (UDCA). 

In addition to secondary BAs, the gut microbiota produces oxo or keto BAs by the oxidation of the hydroxyl groups at ring positions 3, 7 or 12 that are catalyzed by bacteria with hydroxysteroid dehydrogenases (HSDHs). Known bacterial genera involved in such reactions include *Clostridium* groups XIVa, *Eubacterium*, *Bacteroides* and *Ruminococcus* [23]. Other genera such as *Bacteroides*, *Eubacterium* and *Lactobacillus* are known to carry out esterification whereas *Clostridium*, *Fusobacterium*, *Peptococcus* and *Pseudomonas* execute desulfation [24].

A new group of recently uncovered BAs were conjugated to the C24 acyl site, similar to the host conjugation mechanism [25]. As opposed to the conventional amino acids of taurine and glycine, these compounds were conjugated with phenylalanine, leucine and tyrosine on a cholic acid backbone by *Clostridium bolteae*. The precise mechanism of this microbial reconjugation has not yet been resolved. It may rely on a similar mechanism involving a Cys–Asp–His triad with the cysteine acting as the catalytic residue for the nucleophilic attack [26]. Regardless of the mechanism, one could simply suggest that the reconjugation of these residues to the acyl site of BAs would probably influence their chemical properties and thus their signaling functions. Phenylalanine and leucine, two large hydrophobic amino acids, would significantly increase the BA hydrophobicity, which may infer a steric hindrance to any binding mechanism with BA receptors/transporters. The additional hydroxyl group on the aromatic ring of tyrosine may give rise to a few unique properties as it increases the hydrophilicity of the compound and creates a more polar hydrophilic BA, similar to that provided by the host conjugation of taurine to CA. A further investigation is needed to understand how microbes deploy these compounds to impact on the host or competing members of the microbiota.

## 4. Bile Acids and Gut Homeostasis

In addition to their role as emulsifiers that promote fat absorption, BAs may directly target bacterial membranes and cause bacterial damage ranging from envelope and membrane disruption to the complete leakage of intracellular material [27,28,29,30]. Evidence of the direct antimicrobial effects of BAs can be gleaned from mouse models of biliary obstruction that exhibit a significant proliferation of gut microbial communities and increased bacterial translocation [31,32]. These effects can be mitigated by the administration of BAs, which results in the inhibition of bacterial overgrowth. BAs also have indirect antimicrobial effects via FXR-induced antimicrobial peptide production and the FXR-induced regulation of the host immune response [31,33]. 

Significant changes in the gut microbiota composition were reported in rats fed with a primary BA [34]. A CA-supplemented diet resulted in the expansion of Firmicutes, primarily in *Clostridium* spp., whose relative abundance increased from 39% in the controls to 70% in the treated groups [34]. CA and DCA have been also shown to exhibit direct antibacterial effects on *Bifidobacterium breve* and *Lactobacillus salivarius* [35]. Such BAs are likely to contribute to bowel inflammatory injuries [36,37]. On the other hand, secondary BAs, DCA and LCA have been shown to impair *Clostridium difficile* growth in vitro and promote resistance to infection in vivo [38]. 

BAs are likely to influence the gut microbial communities and vice versa, thus highlighting the inter-relationship of the gut microbiota–BA–host axis. Note that the effects of such BAs on the gut microbiota and intestinal epithelium may be inflected by several factors, including: (i) the concentration of BAs (physiological or higher) and the exposure time; (ii) the receptors involved; (iii) the site of inflammation (ileum or colon) and BA transport/absorption processes; and (iv) the host species (whether it is mouse or human). Accordingly, BAs can act in different ways and show either pro-inflammatory effects or anti-inflammatory properties instead.

## 5. Altered Metabolism of Bile Acids and Bile Acid Signaling in IBDs

Earlier studies noted increased levels of unconjugated BAs in both CD and UC patients compared with healthy subjects [39]. Similar results were reported by Rutgeerts et al., where the kinetic of primary BAs showed an increased turnover in patients with ileal dysfunction and the amount of CA fecal loss correlated with the extent of the ileal disease [40]. Higher levels of 7α-hydroxycholest-4-en-3 one (C4), a BA intermediate, were also highlighted in the serum from IBD patients exhibiting a BA malabsorption (BAM) [41]. The severity of BAM in the context of IBDs was increased in the presence of inflammation and after the resection of the distal ileum. In addition, the presence of diarrhea in IBD patients was suggested to be associated with an alteration of specific transport mechanisms within the gut, including those of BAs. A decreased excretion of secondary BAs was detected in UC patients and attributed to a reduced transit time (diarrhea) and fecal pH as well as an impaired 7-alpha-dehydroxylase activity [42,43,44]. Although the fecal BA content was the same in non-relapsing IBDs and healthy subjects, Duboc et al. noted increased levels of conjugated BAs during a disease flare whereas secondary BAs were reduced [10]. Recent metabolomic and metagenomic analyses of stool samples from IBD patients and healthy controls showed a significant increase in primary BAs in patients associated with a lower fecal DCA and LCA [4]. 

Bile acid malabsorption was reported in both CD and UC patients, which may represent a common feature that can be correlated with the severity of the disease [45]. These findings might permit a speculation that a change in the BA pool size is associated with an impaired function of BAs. FXR expression was upregulated by BAs present on the luminal side of epithelial cells but was found to be decreased in the context of inflammation [46]. It is well-known that FXR exerts a role in regulating BA absorption and synthesis by modulating the expression and activation of BA transporters in intestinal epithelial cells such as apical sodium-dependent bile acid transporter (ASBT) [44,47]. A rat model of colitis revealed an increased excretion of BAs in feces and a decreased expression of ASBT in the ileum during the acute phase of colitis [48]. These results indicated the alteration of the expression of ASBT, which plays a key role in maintaining BA homeostasis. Murine studies have demonstrated that BA-regulated FXR modulates intestinal immunity [49,50]. Higher levels of taurine-conjugated BAs were associated with a greater abundance of sulfite-reducing bacterial taxa and colitis in genetically susceptible models [51]. In humans, several studies have described the associations between IBDs, dysbiosis and altered fecal BA profiles [4,10,52]. A reduced expression of FXR was identified in colonic endoscopic biopsies from patients with CD when compared with samples from individuals exhibiting irritable bowel syndrome [50]. 

In addition to FXR, BAs are ligands of TGR5, which is known to mediate ileal and colonic motility [53]. A dysregulated TGR5 expression in mouse models showed an altered intestinal morphology leading to an alteration of the immune response and intestinal permeability [54,55]. Moreover, it was demonstrated that IBD patients presented a decrease in the secondary BA pool that was associated with the alteration of the gut microbiota composition [4,44]. This finding was supported by the fact that germ-free mice presented a significant decrease in secondary BA content [47,56,57]. 

Different BAs are known to have a different potency towards FXR and TGR5 [18]. Accordingly, it is difficult to predict how changes in the gut bacterial composition and, hence, fecal BA composition affect BA signaling and subsequently influence inflammatory processes. LCA and DCA have been shown to suppress pro-inflammatory cytokine production in vitro from human peripheral blood-derived macrophages through the activation of the TGR5 receptor [58]. LCA has recently also been shown to impair Th1 activation, as evidenced by reduced TNFα and IFNγ. This was shown to be mediated through the VDR, a known bile acid receptor, at physiologic concentrations [59]. Recently, DCA was shown to elicit gut dysbiosis, downregulate the FXR-FGF15 axis and promote intestinal inflammation [37].

## 6. Bile Acid Deconjugation in IBDs

**Figure 2:**
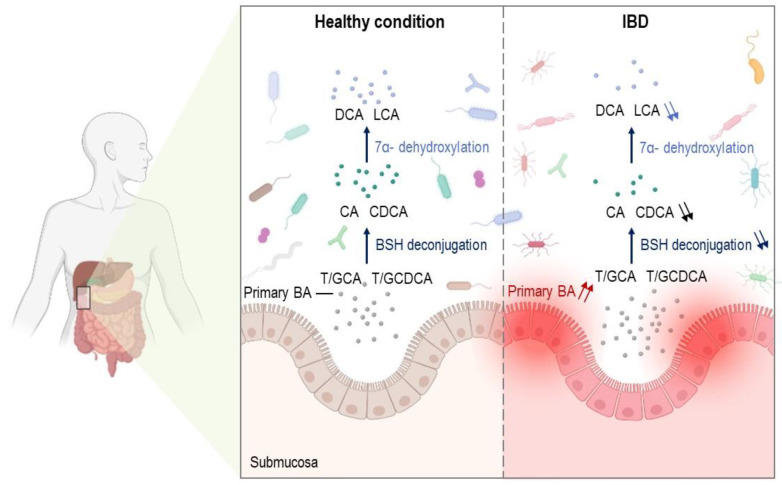
BA profiles in healthy large intestine and IBDs. Under physiological conditions, BAs are metabolized by the gut microbiota through bile salt hydrolase deconjugation and 7α-dehydroxylation into secondary BAs such as DCA and LCA. Abnormal BA biosynthesis and metabolic processes were reported in IBDs with increased primary BA levels, which in turn altered the composition of the gut microbiota and were associated with a lower production of deconjugated and secondary BAs. IBDs: inflammatory bowel diseases; BAs: bile acids; CA: cholic acid; CDCA: chenodeoxycholic acid; T/GCA: taurine/glycine-conjugated cholic acid; T/GCDCA: taurine/glycine-conjugated chenodeoxycholic acid; DCA: deoxycholic acid; LCA: lithocholic acid; BSH: bile salt hydrolase.

Bile salt hydrolase enzymes catalyze the hydrolysis of the amide bond from taurine/glycine residues in conjugated BAs, allowing a further microbial modification of unconjugated BAs [5]. Due to their pivotal role, they are key elements in the balance of BA metabolism [60]. BSH-producing microorganisms are distributed in all major bacterial divisions, most notably in Firmicutes, Bacteroidetes and Actinobacteria [61]. Recently, a significant association between an abundance of *bsh* genes and IBDs was established [62]. The abundance of *bsh* genes belonging to Proteobacteria increased in IBDs whereas Firmicutes decreased in CD. These data are consistent with previous studies that reported a dysbiosis in IBDs characterized by increased levels of Proteobacteria and decreased levels of Firmicutes [63,64,65]. The metabolomic profiling of stool samples from the PRISM cohort (68 CD, 53 UC and 34 non-IBD patients), validated against an independent Netherlands cohort (20 CD, 23 UC and 22 non-IBD subjects), identified BAs as one of the IBD-enriched molecular classes as well as one with the strongest effects in a CD condition [4]. In their study, Duboc et al. demonstrated a link between IBD-associated dysbiosis (including a decrease in bacteria-bearing BSH activity, most notably Firmicutes) and decreased unconjugated and secondary BAs [10] (Figure 2). The dysbiosis observed in IBD patients was characterized by a decrease in the Firmicutes, one of the most potent phyla in terms of BA deconjugation activity [66,67]. By comparing the BA profile of germ-free and conventional mice, a higher proportion of conjugated BAs together with negligible secondary BA levels were observed in germ-free mice compared with conventional mice, thus highlighting the role of the microbiota in the biotransformation of BAs and the loss of function observed in IBD patients [57]. Increased primary and conjugated BAs together with decreased secondary BAs in IBD fecal samples appeared to be consistent across the studies [10,12,68,69]. Despite changes to BAs being observed in other types of samples such as serum, plasma, urine and fasting duodenal bile, a clear trend cannot be identified due to a limited number of studies and their limited consistency [70].

## 7. Other Bile Acid Biotransformations in IBDs

Only a few known bacteria, including all in the *Clostridium Cluster XIVa*, are known to perform the relatively rare 7α-dehydroxylation necessary to convert primary BAs to secondary BAs [71,72]. Of the several proteins involved in secondary BA biosynthesis, one key enzyme is stereo-specific NAD(H)-dependent 3-dehydro-4-bile acid oxidoreductase [73]. A new metagenomics analysis performed by Sinha et al. demonstrated that *bai* genes were expressed in significantly lower levels in UC pouches than in the controls [74]. A deficiency in secondary BAs was also noted in these patients, suggesting their potential anti-inflammatory role. This was mainly ascribed to the abundance of *Clostridium leptum* that was significantly reduced in the IBD fecal samples compared with the healthy subjects [74]. Of particular interest, dysbiosis in IBD patients was also linked to a reduced desulfation activity in the stool [10]. Conversely, higher levels of 3-sulfodeoxycholic acid and chenodeoxycholic acid sulfate were detected in the stools of patients with CD [11]. Likewise, the levels of fecal sulfated BAs were also found to be increased in these patients [11].

## 8. Conclusions

As the prevalence of IBDs has been increasing worldwide, the social and economic burdens related to the disease have been alarming. As such, a better understanding of the underpinning drivers seems of prime importance, particularly because the etiology of IBDs is still largely unexplored. Emerging evidence suggests a potential role of BAs in IBDs. Although BAs have been studied for centuries, recent findings show that we still have much to learn. Bile acids play key roles in intestinal metabolism and cell signaling and are believed to influence the gut microbial composition. In turn, the microbial metabolism of these BAs is known to shape the host physiology. Alterations in BA metabolism and signaling have been shown to influence intestinal homeostasis and drive gut dysbiosis in IBDs, thereby making these molecules an attractive therapeutic target in these diseases. The mechanisms of microbial modifications of BAs continue to be elucidated as do the roles that BA metabolism plays in host health and disease. Bile acids are at the interface of complex molecular interactions between the host and their gut microbiota. The precise impacts of increased levels of BA subtypes associated with IBDs are not yet well-understood. Microbially transformed BAs may be closely intertwined with those of the host and seem to exhibit significant consequences for human physiology. The relevance of these microbial reactions is still largely untapped. The emergence of functional metagenomic tools constitutes a hope to better analyze the role of gut microbiota on BAs in health and disease.
